# Bicuspid Aortic Valve and Endothelial Dysfunction: Current Evidence and Potential Therapeutic Targets

**DOI:** 10.3389/fphys.2020.01015

**Published:** 2020-08-21

**Authors:** Borja Antequera-González, Neus Martínez-Micaelo, Josep M. Alegret

**Affiliations:** ^1^Group of Cardiovascular Research, Pere Virgili Health Research Institute (IISPV), University of Rovira i Virgili, Reus, Spain; ^2^Department of Cardiology, University Hospital Sant Joan de Reus, University of Rovira i Virgili, Reus, Spain

**Keywords:** bicuspid aortic valve, endothelial dysfunction, hemodynamics, endothelial cells, biomarker, CVD, biomarkers, therapeutic target

## Abstract

Bicuspid aortic valve (BAV), the most frequent congenital heart malformation, is characterized by the presence of a two-leaflet aortic valve instead of a three-leaflet one. BAV disease progression is associated with valvular dysfunction (in the form of stenosis or regurgitation) and aortopathy, which can lead to aneurysm and aortic dissection. This morphological abnormality modifies valve dynamics and promotes eccentric blood flow, which gives rise to alterations of the flow pattern and wall shear stress (WSS) of the ascending aorta. Recently, evidence of endothelial dysfunction (ED) in BAV disease has emerged. Different studies have addressed a reduced endothelial functionality by analyzing various molecular biomarkers and cellular parameters in BAV patients. Some authors have found impaired functionality of circulating endothelial progenitors in these patients, associating it with valvular dysfunction and aortic dilation. Others focused on systemic endothelial function by measuring artery flow-mediated dilation (FMD), showing a reduced FMD in BAV individuals. Novel biomarkers like increased endothelial microparticles (EMP), which are related to ED, have also been discovered in BAV patients. Finally, latest studies indicate that in BAV, endothelial-to-mesenchymal transition (EndoMT) may also be de-regulated, which could be caused by genetic, hemodynamic alterations, or both. Different hypothesis about the pathology of ED in BAV are nowadays being debated. Some authors blamed this impaired functionality just on genetic abnormalities, which could lead to a pathological aorta. Nevertheless, thanks to the development of new and high-resolution imaging techniques like 4D flow MRI, hemodynamics has gained great attention. Based on latest studies, alterations in blood flow seem to cause proper modification of the endothelial cells (ECs) function and morphology. It also seems to be associated with aortic dilation and decreased vasodilators expression, like nitric oxide (NO). Although nowadays ED in BAV has been reported by many, it is not clear which its main cause may be. Comprehending the pathways that promote ED and its relevance in BAV could help further understand and maybe prevent the serious consequences of this disease. This review will discuss the ED present in BAV, focusing on the latest evidence, biomarkers for ED and potential therapeutic targets (Figure 1).

## Introduction

### BAV Disease

Bicuspid aortic valve (BAV) is the most frequent cardiac congenital malformation, affecting 1–2% of population, with a higher prevalence in men (3:1; Tzemos et al., 2008; Alegret et al., 2013). It is characterized by the presence of the BAV (two leaflets) instead of a tricuspid one (three leaflets), and it is usually associated with valve dysfunction and aortopathy. BAV is a complex disease that is caused by the abnormal separation of the primordial semilunar valve during embryogenesis, resulting in different phenotypes of the aortic valve (Martin et al., 2015). The most popular classification of BAV is based on the presence/absence of a raphe (type), followed by two supplementary characteristics: the spatial position of the raphe and cusps, as well as the functional status of the valve (Sievers and Schmidtke, 2007). Depending on these phenotypic characteristics of the valve, different outcomes and progression of this disease have been found (Kang et al., 2013).

**Figure 1:**
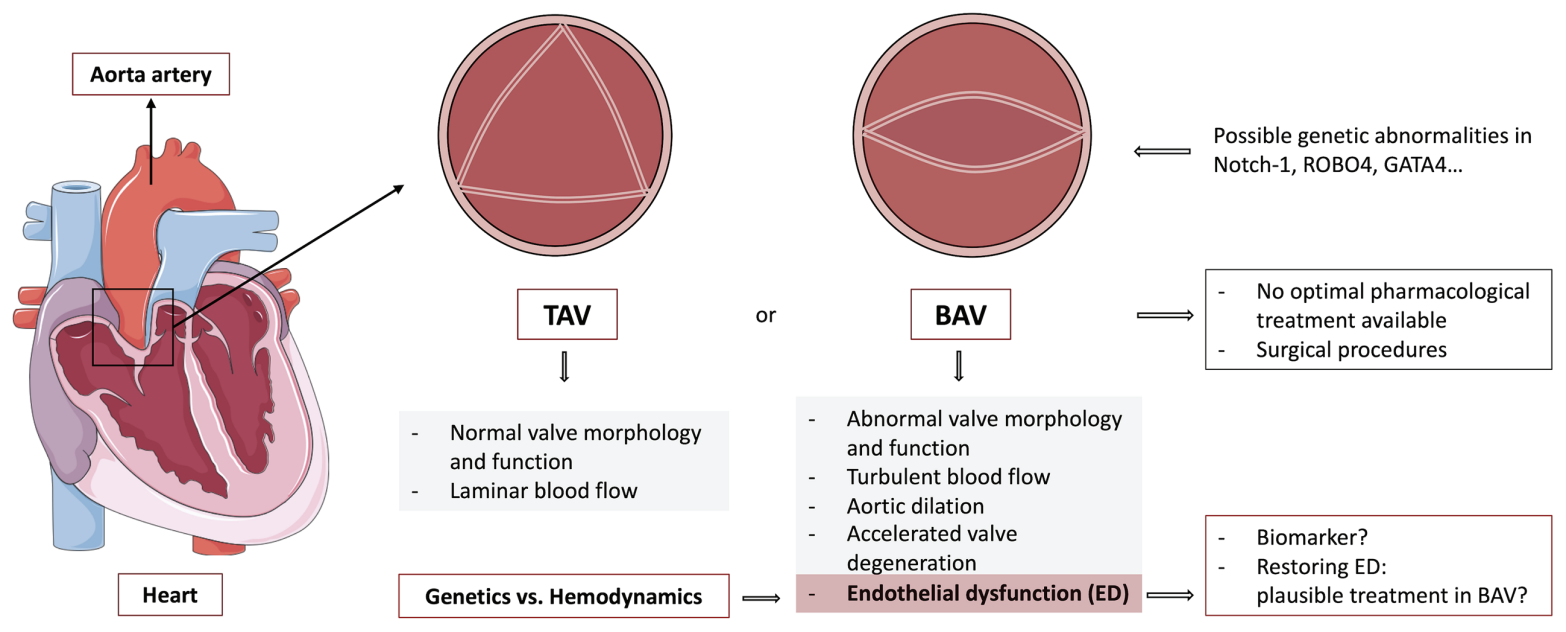
Graphical abstract of the review: a heart is represented at the left, signalizing the aorta, as well as the zone where the aortic valve is found. Different morphologies of this valve are also shown, pointing out the generalities of a healthy tricuspid aortic valve (TAV) and bicuspid aortic valve (BAV) disease. Additionally, endothelial dysfunction (ED) and its importance in BAV disease are also discussed. TAV, Tricuspid aortic valve; BAV, Bicuspid aortic valve; Notch-1, Neurogenic locus Notch homolog protein 1; ROBO4, Roundabout guidance receptor 4; GATA4, GATA binding protein 4. The figure was created by using pictures from Servier Medical Art (http://smart.servier.com/), licensed under a Creative Commons Attribution 3.0 Unported License (https://creativecommons.org/licenses/by/3.0/).

As said before, BAV is a complex disease, which cannot be reduced to just a disorder of valvulogenesis (Siu and Silversides, 2010). The abnormal morphology modifies valve dynamics and promotes eccentric blood flow, which gives rise to alterations of the flow pattern and wall shear stress (WSS) of the ascending aorta. BAV disease is related to ascending aorta dilation (AD), that becomes very frequent and it appears at a much younger age than patients with a normal tricuspid aortic valve (TAV), which can lead to an increased risk of aneurysm formation and aortic dissection (Michelena et al., 2008, 2011; Tzemos et al., 2008, 2010; Alegret et al., 2013). Further, aortic valve dysfunction (stenosis, regurgitation, or both) is frequently observed. Valve dysfunction may be caused by the congenital malformation itself and by the predisposition to accelerated degeneration. In consequence, surgical procedures such as aortic valve replacement and ascending aorta repair in the presence of ascending aorta dilatation may be required throughout the life of these patients (Figure 1).

## Endothelial Dysfunction in BAV Disease: Evidence and Biomarkers

### Endothelium and ED

Recently, evidence of endothelial dysfunction (ED) present in BAV has surfaced (Vaturi et al., 2011; Ali et al., 2014; Alegret et al., 2016; Gavriliuk et al., 2016; Balistreri et al., 2018; van de Pol et al., 2019). ED stands for the abnormal functioning of the endothelial cells (ECs) that cover the inner surface of blood vessels, the endothelium. Although it was considered a rudimentary tissue with basic functions like permeability to water and electrolytes not many years ago (Wilson and Lerman, 2001), numerous functions in which endothelium plays a pivotal role have been identified in the last few years (Kern et al., 1983; Darland and D’Amore, 2001; High et al., 2008; Baeten and Lilly, 2017). It acts as a semi-permeate barrier between blood and the inner portion of the vascular tissue maintaining the homeostasis and regulating the exchange of nutrients, fluids, and macrophages. Endothelium also participates in maintaining vascular tone and it can alter vascular smooth muscle cells (VSMCs) phenotype and differentiation (Kern et al., 1983; Darland and D’Amore, 2001; Ignarro et al., 2001; High et al., 2008; Baeten and Lilly, 2017). Vascular tone is maintained by many vasoconstrictors and dilatators, where nitric oxide (NO) remains as one of the most important vasodilators (Griffith et al., 1984). It is secreted by ECs and it is critical for many physiologic and pathologic processes (Dimmeler and Zeiher, 1999; Garg, 2016). NO is produced by the endothelial nitric oxide synthase (eNOS), present in the cell membrane caveolae (Hecker et al., 1994). When activated, NO diffuses to vascular smooth muscle, causing relaxation by activating guanylate cyclase, which increases cyclic guanosin monophosphate (cGMP), leading to the phosphorylation of vasodilator-stimulated phosphoprotein (VASP) by protein kinase G (PKG) (Arnold et al., 1977; Halbrügge et al., 1990; Elmarakby et al., 2007). VASP facilitates vasodilation by promoting actin elongation and protecting these filaments against capping.

### ED in BAV Disease: Latest Findings

Numerous studies have found associations between eNOS expression, BAV morphology, and aortic dilation (Aicher et al., 2007; Kotlarczyk et al., 2016; Odelin et al., 2019). While some of them indicate a decreased eNOS expression in human BAV tissue in comparison to TAV (Aicher et al., 2007), others focused on concrete aortic regions (Kotlarczyk et al., 2016). One group found out that BAV had higher eNOS expression (both gene and protein) in the R aortic region, which has the greater curvature of the ascending aorta, as well as an increased pSer1177-eNOS (activated eNOS; Kotlarczyk et al., 2016). Nevertheless, they did not find any changes in pSer239VASP, an established biomarker of NO bioavailabity related to endothelial integrity and NO-stimulated soluble guanylate cyclase/cGMP-dependent kinase I (sGC/cGK-I) pathway (Oelze et al., 2000). They hypothesized a possible uncoupled production of NO in favor to produce O_2_, increasing the oxidative stress. Superoxide radicals can react with NO, forming peroxynitrite, a potent oxidant, which causes general ED, as well reducing the bioavailability of NO (Knepler et al., 2001; Peluffo et al., 2009).

Notch signaling pathway is believed to be involved in vascular remodeling and repairing in the cardiovascular system (Kopan and Ilagan, 2009; Balistreri et al., 2018). Endothelial-derived NO increases the expression of Notch signaling target genes in valvular interstitial cells (VICs), protecting these cells and the proper valve from calcification. Neurogenic locus Notch homolog protein 1 (Notch-1) messenger RNA (mRNA) is expressed during valve formation as well as in adult murine aortic valves, leading up to believe that it could participate in the processes of valvulogenesis and the progression of valve disease (Krebs et al., 2000; Garg et al., 2005). Notch-1 represses runt-related transcription factor 2 (Runx2), a regulator of osteoblast cell fate, and it also seems to increase SRY-Box transcription factor 9 (Sox9) expression, which is able to prevent calcification (Garg et al., 2005). It also regulates bone morphogenetic protein 2 (Bmp2), which also seems to take part in calcification prevention (Nigam and Srivastava, 2009). When ED is present, NO expression can be deregulated and calcium deposition may be promoted, leading up to calcific aortic valve disease (CAVD). This calcification could promote valve malfunctioning in form of stenosis and/or regurgitation. Thus, ED seems participate in the first steps in the development of the CAVD (Kostyunin et al., 2019).

Latest researches have focused on the endothelial to mesenchymal transition (EndoMT) and its deregulation in BAV. While EndoMT is essential for pivotal processes, like cardiac development or wound healing, its abnormal activation can lead to different pathologies (Kalluri and Weinberg, 2009). This endothelial transition process starts by losing cell adhesion properties, polarity, and junctions by endocytosis (Le Bras et al., 2012).Later, a restructuration of their cytoskeleton is done in order to become motile and acquire a mesenchymal state. These cells lose typical endothelial markers, like vascular endothelial cadherin (VE-cadherin) or von Willebrand factor (vWF), in order to gain mesenchymal markers, like neural cadherin (N-cadherin) or alpha smooth muscle actin (α-SMA). This process is regulated by several signaling pathways, like transforming growth factor ß (TGF-ß), wingless-related integration site-ß-catenin (WNT-ß-catenin), or Notch (Lamouille et al., 2014). Therefore, alterations in these pathways can deregulate EndoMT. This transition can also be regulated by different micro RNAs (miRNAs) like miR-200 family, which seems to repress the process (Hill et al., 2013). Recent studies have shown deregulation of the EndoMT process in ECs in altered hemodynamics situations too (Muylaert et al., 2016). Others suggest that in BAV, the state of transition of EndoMT is more advanced than in TAV, leading up to a higher EndoMT process, losing essential endothelial characteristics to maintain homeostasis and successful wound healing (Maleki et al., 2019). Furthermore, different studies indicate that EndoMT seems to be more aggressive in dilated BAV. An extensive review of the possible alteration of EndoMT in thoracic aortic aneurysm can be found here (Maleki et al., 2019).

### Biomarkers for ED in BAV Disease

Although nowadays numerous biomarkers for ED have been discovered in BAV (Table 1), there is a lack of a definitive one. For example, while some studies pointed out that asymmetric dimethylarginine (ADMA) has been found to be increased in BAV patients in comparison to TAV patients (Drapisz et al., 2013), others did not find significant differences based just on the valve morphology (Ali et al., 2014). Recent publications have also revealed different miRNAs patterns related to ED in BAV (Martínez-Micaelo et al., 2017; Poggio et al., 2019). In this regard, our group discovered circulating endothelial microparticles (EMPs) – which are related to ED present in BAV patients plasma in higher concentrations than TAV, being also associated with aortic dilatation (Alegret et al., 2016). Recent studies have shown impaired functionality or decreased number of endothelial progenitor cells (EPCs) in BAV (Vaturi et al., 2011; van de Pol et al., 2019). Specifically, one group studieds functionality of peripheral blood endothelial colony-forming cells (pbECFCs) isolated from BAV patients compared to healthy ones as possible a biomarker (van de Pol et al., 2019). They found a decreased migratory capacity in the pbECFCs isolated from BAV patients vs. pbECFCs from healthy controls. Their study also indicated that colony-forming ability typical of these highly-proliferative cells was significantly reduced in pbECFCs isolated from BAV patients with aortic dilatation. Additionally, the systemic effects of ED on regulation of vascular tone has also been evaluated by measuring the brachial artery flow-mediated dilation (FMD; Vogel, 2001) in BAV patients, and these studies have also concluded that BAV patients have a lower FMD when compared to healthy ones (Tzemos et al., 2010; Ali et al., 2014; Wang et al., 2018).

**Table 1:** Summary of the ED biomarkers discovered in BAV disease.

	Biomarker	Findings in BAV	References
Plasma biomarkers	ADMA	Higher levels of ADMA-inhibitor of eNOS have been found in some BAV patients	Drapisz et al. (2013); Ali et al. (2014)
	EMPs	Higher concentrations of EMPs has been associated with BAV and, specifically, aortic dilation	Alegret et al. (2016)
	miRNAs	Diverse miRNAs patterns have been identified to be related to ED in BAV	Martínez-Micaelo et al. (2017); Poggio et al. (2019)
ECs assays	EPCs	Impaired functionality and/or decreased number of circulating EPCs in BAV	Vaturi et al. (2011)
	ECFCs	Impaired functionality and/or decreased number of circulating ECFCs in BAV	van de Pol et al. (2019)
Non-invasive	FMD	Lower levels of FMD are related to BAV and its aortopathies	Tzemos et al. (2010); Ali et al. (2014); Wang et al. (2018)

BAV, bicuspid aortic valve; eNOS, endothelial nitric oxide synthase; ECs, endothelial cells, ADMA, asymmetric dimethylarginine; EMPs, endothelial microparticles; miRNAs, micro RNAs; EPCs, endothelial progenitor cells; ECFCs, endothelial colony forming cells; FMD, flow-mediated dilation.

Therefore, it is hard to deny that ED is probably directly related to BAV and its worse prognosis, especially with aortic dilatation and aortic stenosis (Vaturi et al., 2011; Ali et al., 2014; Alegret et al., 2016; Gavriliuk et al., 2016; Balistreri et al., 2018; van de Pol et al., 2019). Nevertheless, the pathways that could promote ED and its relevance in BAV are not fully known. Additionally, it is not yet fully elucidated if this malfunctioning is due to a genetical predisposition of these ECs to dysfunction, or it is caused by the proper alteration of hemodynamics that modifies ECs phenotype and function. Thus, further ED research in BAV should be assessed in order to concrete the specific molecular pathways that take place in this complex disease.

## Genetics and ED in BAV Disease

While BAV malformation may be caused by genetic abnormalities that occur sporadically, with familiar clustering in some cases (Gale et al., 1977; Brown et al., 2003), the main cause of associated ED and aortopathies in BAV, as well as the pathways involved, are still a topic of discussion.

### Genetic Abnormalities in BAV Disease Related to ED

While there is much controversy between data related to BAV associated aortopathies and their first-degree relatives (FDRs), one group found significant differences in ED biomarkers in FDRs of BAV compared to FDRs of TAV individuals (Li et al., 2020). Further studies with a larger sample size and definitive ED biomarkers should be performed in order to make definitive conclusions. Accordingly, one group performed a genome-wide association study (GWAS) comparing BAV vs. control patients (Fulmer et al., 2019). They found out that a variety of single nucleotide polymorphisms (SNPs) in Exocyst-Cilia Machinery system could be the origin of the BAV phenotype. This recent study also indicates that removal of exocyst complex component 5 (Exoc5), specific of the endocardium, was enough to cause BAV malformation and promote calcified aortic valve stenosis in adulthood. This fact could support the genetic hypothesis of BAV phenotype and the presence of deregulation of essential processes in ECs, possibly endorsing ED.

**Figure 2:**
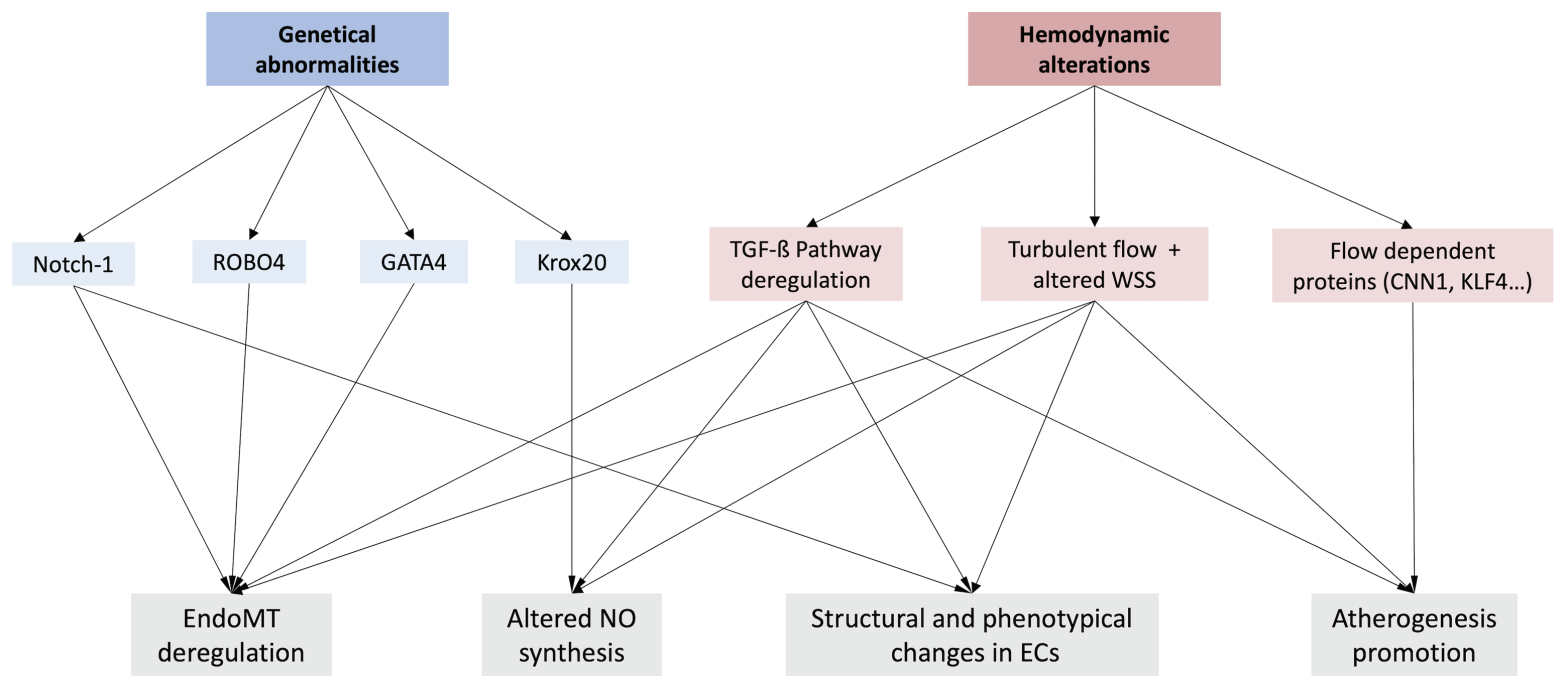
Representation of the two main hypothesis about a possible ED in BAV disease (genetical abnormalities and/or hemodynamic alterations), as well as some of the most relevant phenomenon these variations may provoke. Notch-1, Neurogenic locus Notch homolog protein 1; ROBO4, Roundabout guidance receptor 4; GATA4, GATA binding protein 4; Krox20, Early growth response 2; EndoMT, Endothelial-to-mesenchymal transition; NO, Nitric oxide; TFG-ß, Transforming growth factor ß; WSS, Wall shear stress; CNN1, Calponin-1; KFL4, Krüppel-like Factor 4.

Numerous studies have found relation between BAV disease, associated aorthopaties, and mutation in components of Notch pathway (Garg et al., 2005; High et al., 2008; Wang et al., 2015). Furthermore, Notch expression has been found to be deregulated in adults ECs of some BAV patients (Balistreri et al., 2018). Other genetic mutations are known to cause a variety of BAV phenotypes, as well as their associated aortopathies (Park et al., 2003; Pu et al., 2004; Odelin et al., 2019). One example of genetical abnormality, which can result in BAV is roundabout guidance receptor 4 (ROBO4), a specific vascular receptor that prevents ECs to migrate (Park et al., 2003). This study indicated that ROBO4 expression is significantly decreased in ECs of some BAV patients. Mutations of ROBO4 seem to promote deregulation of EndoMT, making endothelium lose its typical barrier function, as it reduces tight junction protein 1 (TJP1) and VE-cadherin expression, decreasing adherent and tight junctions in ECs (Maleki et al., 2019). This group found that in homozygotic null ROBO4 mice, BAV disease, as well as valve stenosis, was occasionally present. Genetical abnormalities in GATA binding protein 4 (GATA4) gen, which is a well-known regulator or cardiac morphogenesis (Pu et al., 2004), have also been found recently in BAV patients. Furthermore, some studies indicated that this alteration significantly impaired the EndoMT process, a critical step in heart valve development (Yang et al., 2017). Additionally, they found that these differences in expression were not significant when comparing BAV patients with or without AD. These findings suggest that this genetic abnormality could maybe not be the only cause of related aortopathies and ED in these BAV patients (Yang et al., 2017). Similar to GATA4, endothelial GATA binding protein 5 (GATA5) null-mice also results in a BAV morphology (Laforest et al., 2011). Another example of genetic mutation that promotes BAV malformation involves early growth response 2 (Krox20), which has also been found to be mutated in some BAV patients (Odelin et al., 2019). Krox20 binds to the promoter of nitric oxide synthase 3 (Nos3), promoting the expression of Nos3. These mutations could decrease Nos3 expression and therefore NO, deregulating vasodilation and Notch signaling pathway, both critical in maintaining vascular health (Odelin et al., 2019). Although different genes can be discussed, all these mutations are related to an incorrect endothelial function, and their deregulation can be related to ED (Figure 2).

In brief, numerous genetical abnormalities are known to cause BAV malformation and related aortopathies. BAV is believed to have an autosomal dominant inheritance pattern with incomplete penetrance and variable expressivity (Huntington et al., 1997; Braverman et al., 2005). Some of these mutations also seem to provoke ED by altering their proliferation-apoptosis equilibrium, deregulating EndoMT process, reducing their ability to act as a barrier, modifying their phenotype, etc (Maleki et al., 2019). ED is nowadays thought to be directly related to progression of BAV disease, as we have discussed before. Nevertheless, there has not been found yet a common genetic abnormality between all BAV patients. Diverse genetic mutations have been found in different BAV patients, bearing out the complexity and heterogeneity of this disease. Moreover, patients with different outcomes or not found genetic alterations that cause BAV are also present. Therefore, it seems like even though genetical predisposition can easily play an important role, it may not be the only one. Finally, the consensus of a definitive and trustworthy ED biomarker should be assessed in order to validate these findings.

## Hemodynamics in BAV Disease: Is it the Key Player?

Until quite recently, the most accepted theory about aortic dilation was based on the existence of a genetical predisposition which resulted in BAV disease and associated aortopathies. Nevertheless, the heterogeneity of this disease makes hard to believe that only genetics could play a role in BAV. Furthermore, the development of new advances in imaging techniques has opened a topic on discussion about how alterations in blood flow could affect the endothelium and the vascular system. Specifically, in BAV, abnormal valve opening usually leads to an eccentric and turbulent blood flow in ascending aorta.

### Blood Flow and ECs

Endothelium homeostasis is mostly maintained by the laminar blood flow that ECs are exposed. In laminar flow areas, ECs produce beneficial factors for their proper survival, quiescence, and barrier function. In these areas, coagulation, proliferation of VSMCs and leucocyte extravasation is suppressed (Reinhart-King et al., 2008). Nevertheless, in turbulent blood flow areas where shear stress is altered, their phenotype is modified, increasing the permeability of the endothelium, deregulating proliferation-apoptosis equilibrium and enhancing adhesion properties for monocytes (Zhou et al., 2014). Normally, ECs phenotype varies largely in the endothelium depending on the place these cells are covering due to different blood flow pattern, showing heterogeneity across vascular beds (Aird William, 2007). These cells have a variety of mechanical receptors, which can perceive alterations in blood flow hemodynamics and produce different biological responses. One of the most studied mechanotransducers in ECs is a complex composed by platelet endothelial cell adhesion molecule-1 (PECAM-1), VE-cadherin, vascular endothelial growth factor receptor (VEGFR) 2 and 3 (Osawa et al., 1997; Tzima et al., 2005; Conway et al., 2013). Blood flow increases tension of PECAM-1, triggering the association of this protein with vimentin cytoskeleton (Conway et al., 2013). This tension promotes the activation of a proto-oncogene tyrosine-protein (Src) kinase, leading up to the transactivation of VEGFR and the activation of phosphatidylnositol 3-Kinase (PI3k) and eNOS, which in turn products NO in order to induce vasodilation (Fleming et al., 2005; Tzima et al., 2005). Integrins are also activated by PI3K, which are involved in cell alignment in laminar flow. When turbulent flow is present, tissue remodeling is active and inflammatory pathways are activated (Buschmann et al., 2003). This process seems to be controlled by blood flow, suppressing it when there is no blood flow alteration, and activating it when there are changes in hemodynamics. A complete review about shear stress involved in vascular remodeling can be found here (Baeyens et al., 2016).

ECs can translate biomechanical forces into specific responses. When endothelium is intact, it regulates VSMCs proliferation by endothelial heparan sulfate proteoglycans (HSPG), controlled by mechanosensitive pathways like transforming growth factor beta (TGF-β; Ettenson et al., 2000). Nevertheless, when hemodynamics and shear stress alterations are present, as in BAV, deregulations in these pathways provoke proper VSMCs growth deregulation (Baker et al., 2008). TGF-β induction by shear stress seems to be related to the activation of krüppel like factor 2 (KLF2) signaling cascade, which is essential for maintaining the correct endothelial functioning (Baker et al., 2008; Walshe et al., 2013). Among others, KLF2 induces eNOS activity and production, which could be translated to an increased NO production, essential to maintain vascular tone and the endothelium (Fontijn et al., 2015). As said before, TGF-β also plays a key role in the EndoMT process (Ten Dijke et al., 2012). Therefore, its deregulation by alteration in hemodynamics could induce proper endothelial phenotypic changes, modifying the endothelium and homeostasis. This pathway deregulation seems to contribute to induce BAV related aortopathies like aortic aneurysm (Angelov et al., 2017; Rocchiccioli et al., 2017).

### Hemodynamics Alterations in BAV Disease

Recently, numerous studies have reported that changes in flow characteristics and WSS are present in BAV disease, and these alterations directly affect the endothelium (Meierhofer et al., 2013; Baeyens et al., 2016; Chistiakov et al., 2017). Thanks to development of new and high resolution imaging techniques like time-resolved three-dimensional phase contrast MRI (Kozerke et al., 2001; Frydrychowicz et al., 2007) also known as 4D flow MRI, hemodynamics have gained attention in the BAV field. This technique allows quality and quantitative flow measurement, like flow velocity, flow direction, wall shear stress, complex flow patterns, etc (Sträter et al., 2018). The progress of development of new imaging techniques in the cardiovascular field has also helped acquire more fast and accurate data in order to obtain prognosis or diagnosis of several diseases. Specially, in BAV, it has helped further understand the hemodynamics alterations produced by morphological abnormalities of the aortic valve. Recently, it has been described that hemodynamics depends directly on BAV phenotype (Rodríguez-Palomares et al., 2018). Several studies have also shown differences between the arterial wall of BAV and TAV patients, mostly at the intimal area (Meierhofer et al., 2013). It has also been indicated that regions with altered WSS are more likely to extracellular matrix dysregulation and elastic fiber degeneration, possibly inducing aortic dilation (Bissell et al., 2013; Meierhofer et al., 2013; Guzzardi et al., 2015). Others have shown that blood flow alterations are capable of promoting medial degeneration by metalloprotease pathways (Atkins and Sucosky, 2014; Andreassi and Della Corte, 2016). Furthermore, when comparing the areas of the artery with the highest impact of blood flood (jet) and more laminar flow in the artery between these BAV patients and TAV patients, noticeable differences were found (Grewal et al., 2019). Thickening of the intimal in the jet area was observed when compared to another area with less turbulent flow. This study also linked this thickening with an increased cobblestone morphology of ECs, indicating that alterations in hemodynamics can affect ECs morphology. Nevertheless, this study did not profound on the possible EndoMT ongoing. Others did focus on EndoMT in BAV and found that hemodynamics can deregulate this process that is believed to cause loose of elasticity in arterial walls and therefore, aortic dilation (Mahler et al., 2014; Moonen et al., 2015).

### Flow-Dependent Proteins

In BAV, the expression of some flow-dependent proteins has been found to be altered. A study indicated the influence of hemodynamics alterations in the expression of calponin 1 (CNN1), which is induced in turbulent flow areas in ECs (Hsu et al., 2019). CCN1 binds to its receptor, activating nuclear factor kappa B (NF-kB), which promotes ED and atherogenesis. It is also been observed that laminar blood flow and high shear stress makes ECs more resistant to oxidation by promoting autophagy. However, this effect can be attenuated by the presence of turbulent blood flow and altered shear stress (Liu et al., 2015). Another example of flow-dependent protein would be krüppel-like factor 4 (KLF4), which levels are also known to be decreased in ECs at the jet impacts areas due to turbulent flow. This protein contributes to regulate inflammation and it has atheroprotective characteristics (Jiang et al., 2014). Hence, any of these alterations could deregulate endothelium homeostasis, promoting ED, and in consequence, an increased risk of aortic dilation. Additionally, our group found increased levels of EMPs in BAV patients with aortic dilation compared to healthy subjects (Alegret et al., 2016). These microparticles are related to endothelial damage and their expression is inversely correlated with WSS (Vion et al., 2013). Therefore, changes in hemodynamics which promote an eccentric flow in BAV could alter EMPs levels as a sign of ED (Figure 2).

Although lots of evidence from hemodynamics importance has emerged lately in the BAV field, some studies pointed out that it could be not the only factor affecting aorthopaties and ED in BAV patients. While some genetic alterations in many key genes that participate in pivotal pathways in ECs are found in some BAV individuals, the genetical pathology of most of them remains unknown. Lately, researchers have focused on hemodynamics alterations as the main cause of associated pathologies in BAV. Although using a small cohort of patients, one group made a point in favor of this hypothesis, indicating that aortic dilatation progression in BAV patients who underwent valve replacement became similar to TAV patients (Regeer et al., 2016). This could mean hemodynamics alterations could play a key role in development of BAV associated aortopathies. As explained before, most of the recent evidence shows that alteration of blood flow is the fundamental key to ED, kicking off the process of degeneration of the aortic media. However, some experimental studies in BAV patients without significant alterations in blood flow yet severe aortopathies that have focused on genetic predisposition, suggesting that it may also play a complementary role.

## ED as a Potential Biomarker and Therapeutic Target in BAV Disease

Based on the latest evidence, ED might be related to BAV and its associated aortopathies (Vaturi et al., 2011; Ali et al., 2014; Alegret et al., 2016; Gavriliuk et al., 2016; Balistreri et al., 2018; van de Pol et al., 2019). While the mechanisms and pathways involved in this process are not fully elucidated, ED seems to be linked to the progression of BAV disease, leading up to aortopathy and valvular dysfunction. Therefore, it seems reasonable that ED could be used as an additional general prognosis biomarker of valvular dysfunction and related aortopathies in BAV. Many methods that are currently used for measuring ED could be useful: FMD, plasma biomarkers, functionality assays of cultured ECs “*in vitro*,” miRNA patterns, EMPs, etc. In any case, we strongly believe that ED should be further studied in BAV in order to concrete its importance as well as its related signaling pathways.

### Potential Therapeutic Targets for ED in BAV Disease

If ED is related to BAV aortopathies and its worse prognosis, it could be hypothesized that restoring a correct endothelial function could at least help slow down BAV aortopathy progression. Thus, a variety of potential treatments could be considered in order to handle ED in BAV disease.

Nowadays, stem cell therapy and gene therapy are a reality in many disease treatments, and they are showing promising results, as well as, health safety. Many trials have been performed in some diseases that involve ED, like peripheral artery disease (PAD; Frangogiannis, 2018; Paschalaki and Randi, 2018). In this field, we should talk about endothelial progenitor cells (EPCs). EPCs can be classified in early endothelial progenitor cells (eEPCs) and late endothelial progenitor cells, or most commonly called endothelial colony forming cells (ECFCs; Fadini et al., 2012). The first subgroup (eEPCs) seems to act preferably in a paracrine way, secreting numerous angiogenic factors, like VEGF (Yang et al., 2015). On the other hand, ECFCs are highly proliferative and can promote angiogenesis *in vitro* and *in vivo* and are involved in wound healing (Fadini et al., 2012). A recent study has found that ECFCs in BAV have less clonogenic potential in BAV with dilated aorta (van de Pol et al., 2019). ECFCs cell therapy could help restore correct endothelial function and maybe attenuate disease progression (Banno and Yoder, 2018). ECFCs can be obtained from peripheral blood (Ingram et al., 2004) or white adipose tissue (Lin et al., 2013), which makes easy to obtain them. Allogenic cell therapy could be easily performed or even autologous cell therapy. ECFCs can even be cultured and pre-activated “*in vitro*” before injection in order to increase their yet great potential (Rosova et al., 2008; Mena et al., 2014, 2018). Nowadays, combined cell therapy is also used and it seems to have great results (Banno and Yoder, 2018). Additionally, if severe genetic abnormalities are present in patients ECs, gene editing could be performed before injecting them again or using allogenic therapy should be considered (High and Roncarolo, 2019). Nevertheless, until date, no cell therapy has been performed in BAV patients that we have found. The possible benefit of this therapy would be in line with which was the main cause of ED. It also must be said that if associated ED in BAV is mainly due to an alteration in hemodynamics, cell therapy may not be the optimal treatment, as the blood flow would remain altered, and therefore, it could continue promoting ED.

Pharmacological treatment may also be analyzed as a way of rescuing correct endothelial functioning in BAV disease. Numerous drugs classically used as standard treatment in cardiovascular diseases might slightly influence endothelial function: angiotensin-converting enzyme 1 (ACE1) inhibitors, statins, beta blockers, antioxidants, etc.; yet there is a lack of definitive clinical evidence (Su, 2015). Specifically, diverse studies have shown that pitavastatin seems to significantly improve FMD (Katsiki et al., 2018). Other studies suggest that Rac family small GTPase 1 (Rac1) could ameliorate human endothelial function in veins, as well as reducing oxidative stress, in a NO-dependent manner (Carrizzo et al., 2017); becoming an interesting target for improving ED in BAV disease.

Finally, a silencing therapy for specific miRNAs related to ED in BAV with antisense oligonucleotides could also be an interesting approach (Lima et al., 2018). As we explained before, different miRNAs patterns related to ED have been observed in BAV. Thus, silencing these specific ED-related miRNAs may help restoring endothelial function, as well as slowing BAV disease progression (Martínez-Micaelo et al., 2017; Poggio et al., 2019).

Nevertheless, if the theory that ED is mainly related to alterations in hemodynamics in the aortic flux due to BAV abnormal morphology was considered, it would be questionable to believe the possibility of a completely successful treatment while the altered blood flow remains intact. While the only definitive treatment to redirect blood flow comprise replacing or repairing the BAV nowadays, this option is not always recommended as it involves inherent intervention risks, as well as potential long-term complications of the repaired or new valve (Tillquist and Maddox, 2011; Nishimura et al., 2014; Baumgartner et al., 2017).

## Discussion

In summary, evidence of ED in BAV has surfaced in the latest years, showing a direct relation with BAV related aortopathies and worse prognosis of BAV. Further research should be performed in order to understand the pathways involved in this BAV related ED. New advances in imaging techniques showed an important effect of alterations in hemodynamics in ED, although a genetical predisposition should also be considered. In both cases, endothelium seems to be altered and ED might be promoted. As discussed before, specific biomarkers for ED could be powerful biomarkers for studying BAV prognosis, as well as its related aortopathies. Additionally, we hypothesize that restoring correct endothelial functioning could maybe help slow down BAV disease progression, mainly in patients where the blood flow is not extremely altered. Further studies focused on improving endothelial functioning in BAV patients should be performed in order to assess its utility and importance in this complex and heterogeneous disease.

## Author Contributions

BA-G as the first author, wrote the major part of this paper, as well as searching for bibliography and unifying latest reports about any kind of endothelial dysfunction in bicuspid aortic valve. NM-M and JA helped in the writing process, supervised the manuscript and approved it. All authors contributed to the article and approved the submitted version.

### Conflict of Interest

The authors declare that the research was conducted in the absence of any commercial or financial relationships that could be construed as a potential conflict of interest.
